# Genome-wide analysis of DNA methylation and gene expression defines molecular characteristics of Crohn’s disease-associated fibrosis

**DOI:** 10.1186/s13148-016-0193-6

**Published:** 2016-03-12

**Authors:** Tammy Sadler, Jeffrey M. Bhasin, Yaomin Xu, Jill Barnholz-Sloan, Yanwen Chen, Angela H. Ting, Eleni Stylianou

**Affiliations:** Department of Pathobiology, Cleveland Clinic Lerner Research Institute, 9500 Euclid Avenue/NC-22, Cleveland, OH 44195 USA; Department of Molecular Medicine, Cleveland Clinic Lerner College of Medicine at Case Western Reserve University, Cleveland, OH USA; Genomic Medicine Institute, Lerner Research Institute, Cleveland Clinic, 9500 Euclid Avenue/NC-22, Cleveland, OH 44195 USA; Department of Biostatistics, Vanderbilt University School of Medicine, Nashville, TN USA; Institute for Computational Biology, Case Western Reserve University, Cleveland, OH USA; Department of Gastroenterology and Hepatology, Digestive Diseases Institute, Cleveland Clinic, Cleveland, OH USA

**Keywords:** DNA methylome, Transcriptome, Intestinal fibrosis, Next generation sequencing, RNA seq, Omics, Crohn’s disease, Inflammatory bowel disease

## Abstract

**Background:**

Fibrosis of the intestine is a common and poorly understood complication of Crohn’s disease (CD) characterized by excessive deposition of extracellular matrix and accompanied by narrowing and obstruction of the gut lumen. Defining the molecular characteristics of this fibrotic disorder is a vital step in the development of specific prediction, prevention, and treatment strategies. Previous epigenetic studies indicate that alterations in DNA methylation could explain the mechanism by which mesenchymal cells adopt the requisite pro-fibrotic phenotype that promotes fibrosis progression. However, to date, genome-wide analysis of the DNA methylome of any type of human fibrosis is lacking. We employed an unbiased approach using deep sequencing to define the DNA methylome and transcriptome of purified fibrotic human intestinal fibroblasts (HIF) from the colons of patients with fibrostenotic CD.

**Results:**

When compared with normal fibroblasts, we found that the majority of differential DNA methylation was within introns and intergenic regions and not associated with CpG islands. Only a low percentage occurred in the promoters and exons of genes. Integration of the DNA methylome and transcriptome identified regions in three genes that inversely correlated with gene expression: wingless-type mouse mammary tumor virus integration site family, member 2B (*WNT2B*) and two eicosanoid synthesis pathway enzymes (prostacyclin synthase and prostaglandin D2 synthase). These findings were independently validated by RT-PCR and bisulfite sequencing. Network analysis of the data also identified candidate molecular interactions relevant to fibrosis pathology.

**Conclusions:**

Our definition of a genome-wide fibrosis-specific DNA methylome provides new gene networks and epigenetic states by which to understand mechanisms of pathological gene expression that lead to fibrosis. Our data also provide a basis for development of new fibrosis-specific therapies, as genes dysregulated in fibrotic Crohn’s disease, following functional validation, can serve as new therapeutic targets.

**Electronic supplementary material:**

The online version of this article (doi:10.1186/s13148-016-0193-6) contains supplementary material, which is available to authorized users.

## Background

Intestinal fibrosis is a devastating complication of Crohn’s disease (CD), a major type of inflammatory bowel disease (IBD) [1]. Characterized by a chronic transmural inflammation of the intestine, CD is disabling, incurable, and of unknown etiology. The associated fibrosis comprises prolonged abnormal wound repair and tissue remodeling leading to excessive deposition of a collagen-containing extracellular matrix (ECM). Hypertrophy of the submucosa and muscularis is a major contributor to the increased rigidity and thickness of the bowel wall. These changes typically lead to stricture formation and fibro-stenosis, a major cause of serious complications and surgical procedures in CD patients. In the absence of fibrosis-specific drugs, anti-inflammatory therapies have not prevented or reduced the incidence of fibrosis. For the CD patients that succumb to this complication, surgical intestinal resection is currently the only treatment option and provides temporary symptomatic relief without cure or alteration of disease progression [2]. In this context, the ability to predict the patients that develop fibrosis remains an important challenge that would significantly improve the clinical management of IBD.

A number of factors have been proposed to have a role in the etiology of CD [1, 3, 4]. These include genetic susceptibility, defects in innate immunity, undefined environmental factors, and alterations in the microbiome. Recent genome-wide association studies appear to explain only a minority of the risk associated with development of CD [5, 6]. The high rate of discordance among monozygotic twins and the increased prevalence of CD over recent decades suggest environmental factors may be at play. As epigenetic changes are dynamically responsive to the environment, they are likely to play a key role in the pathogenesis of fibrosis and to offer a molecular explanation for how the intestine becomes pro-fibrotic.

DNA methylation is the addition of a methyl group to the 5-position of the DNA base cytosine. Genome-wide changes in DNA methylation have been shown to be major contributors to cancer, mammalian development, gene transcription, and phenotype in a range of diseases [7, 8], including a variety of autoimmune and inflammatory conditions. Recent epigenetic profiling of IBD patients has comprised DNA methylome signatures of colon tissue, whole blood, and B cells for both major types of inflammatory bowel disease: ulcerative colitis (UC) and CD [9–13]. Of the few previous studies, two of these correlated changes in DNA methylation with gene expression [12, 13] and only one analyzed a purified (B cells) cell type [10]. Furthermore, of the three previous studies that have analyzed epigenetic changes in intestinal fibrogenesis, none of these defined changes in DNA methylation. One, from our lab, showed that chromatin modifications are linked with activation of type I collagen gene expression in endothelial to mesenchymal transition [14], a feature of intestinal fibrosis in vivo*.* The two other labs focused on specific miRNAs in the fibrotic intestine [15, 16]. Moreover, all published studies to date of the DNA methylation in fibrotic diseases have been limited to studying restricted subsets of genes or to the use of microarrays that lack genome-wide coverage [17–23]. We report here the use of an unbiased, genome-wide approach to define the DNA methylome and the transcriptome of fibroblasts isolated from colons of control and CD patients. This approach avoids the issues of heterogeneity of tissue/biopsy samples and employs next generation sequencing using the methyl-CpG binding domain (MBD) of MBD2, called MBD-isolated genome sequencing (MiGS). MiGS is based on the capacity of MBD2 to bind with high affinity and specificity to DNA containing densely methylated cytosines [24]. A sequencing library heavily enriched for these methylated sequences within sheared genomic DNA is coupled to next generation sequencing so that the location of DNA methylation at specific genomic loci can be quantified and compared. We integrated this information with RNA sequencing (RNA-seq) data to identify key molecular interactions that lead to fibrosis pathology. Our identification of key differentially methylated regions (DMRs) in intestinal fibrosis provides new molecular characteristics for fibrostenotic CD and a resource for studying epigenetic mechanisms that could help classify different stages of fibrosis and identify patients predisposed to developing this major complication of IBD.

## Results

### Genome-wide changes in DNA methylation in fibrotic human intestinal fibroblasts

**Fig. 1:**
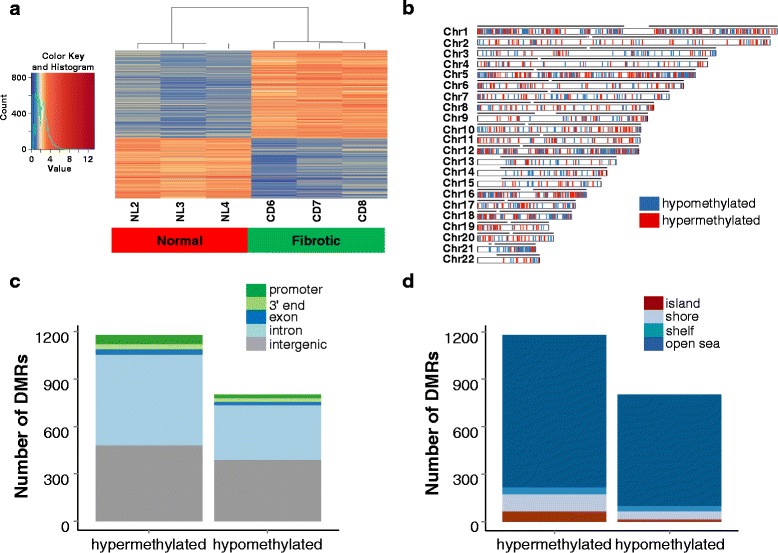
Genome-wide differentially methylated regions (DMRs) in fibrotic human intestinal fibroblasts (HIF). **a** Heatmap of read counts at all sharp yes/no DMRs. Square root transformed read counts were plotted for each detected DMR. Higher read counts (*red*) indicate stronger evidence for the presence of methylation. Lower and zero read counts (*blue*) indicate absence of methylation. Each row represents one DMR. Read counts have been normalized to the number of 50-bp windows in each DMR. **b** Karyogram showing genome-wide coverage of differentially methylated regions (DMRs). Hypermethylated (*red*) and hypomethylated (*blue*) regions in Crohn’s disease HIF when compared to normal controls are shown. *Black lines* above each chromosome represent regions covered by sequencing reads to show genome-wide coverage. **c** The proportion of DMRs that overlap with promoters, gene 3′ ends, exons, introns, and intergenic regions is shown. RefSeq genes were used to define transcription units. **d** The number of sharp yes/no DNA methylated loci in CpG islands versus CpG shores (2000-bp flanking CpG islands), shelves (2000-bp regions flanking shores), and open sea regions (loci greater than 4 kb from CpG islands) is shown

Genome-wide DNA methylation profiles were generated from human intestinal fibroblasts (HIF) isolated from colon resection specimens that were either normal or from patients with fibrostenotic CD. We identified statistically significant regions of differential DNA methylation between the two groups at a false discovery rate (FDR) threshold of <5 %. Both quantitative and qualitative differences in DNA methylation were detected (Additional file 1). Qualitative differences were defined as sharp yes/no DNA methylation with a clear presence or absence of DNA methylation between the normal and fibrotic groups. In sharp yes/no DMRs, one group has zero or statistically near-zero sequencing reads, indicating a lack of methylation, whereas the other group shows a strong enrichment of reads, indicating the presence of methylation. Heatmap visualization and hierarchical clustering of the sharp yes/no DMRs showed a striking difference between the DMRs in normal and CD samples (Fig. 1a). These sharp yes/no DMRs were detected throughout the genome on all 22 autosomes (Fig. 1b). Of the sharp yes/no DMRs, 1180 DMRs represent hypermethylation in the fibrotic samples as compared to the normal samples, and 802 represent hypomethylation. We focused further analysis on qualitative differences in methylation because the quantitative differences (where both conditions are statistically different and have high numbers of reads) are likely methylated in all conditions [25].

### Genomic and CpG island context of differential DNA methylation in intestinal fibrosis

Evidence from recent studies suggests that the genomic location of DNA methylation is a major contributor to the type of function performed by this epigenetic modification [7, 8]. Based on the global distribution of the hyper- and hypo-DNA methylated loci, we determined the frequency of loci in promoter, inter-, and intragenic regions (Fig. 1c and Additional file 1). Promoter regions were defined as +1000 bp to −500 bp relative to transcription start sites, 3′ end gene regions as +1000 bp to −1000 bp of transcription termination sites, and intergenic regions as the remaining regions of the genome. The NCBI reference sequence database (RefSeq) was used to define the transcription units for all genes. In the cases where DMRs were located in multiple gene regions, the overlaps were prioritized as follows: promoter > 3′ end > exon > intron > intergenic. Only a minor percentage of the mapped differential DNA methylation occurred in promoters (5.0 and 2.7 % for hypermethylated and hypomethylated DMRs, respectively), and a large percentage occurred in intergenic regions (40.8 and 48.4 %). DNA methylation within gene bodies has also been reported to have an important role in transcriptional control [26]. While only a small percentage of DMRs overlapped with annotated exons (3.0 and 2.9 %), DMRs were abundant within introns (48.6 and 43.1 %).

We also asked whether methylated promoter CpG islands (CGIs), associated with repression of gene transcription [27], are a feature of intestinal fibrosis (Fig. 1d). The vast majority of differential DNA methylation was found outside CGIs. For hypermethylated and hypomethylated DMRs, respectively, only 5.9 and 1.9 % were in CGIs, 9.7 and 6.4 % in shores (2000-bp flanking CpG islands), and 4.0 and 4.1 % in shelves (2000-bp flanking shore regions). Of all the DMRs, the open sea regions (loci greater than 4 kb from CpG islands) contained most of the differential non-CpG island methylation (86.3 and 87.7 %). Published data indicate that alterations in DNA methylation outside CGIs in shelves/shores can play a role in gene transcription and may be cell-type specific [28].

### Genome-wide transcriptome analysis of fibrotic and normal intestinal fibroblasts

**Fig. 2:**
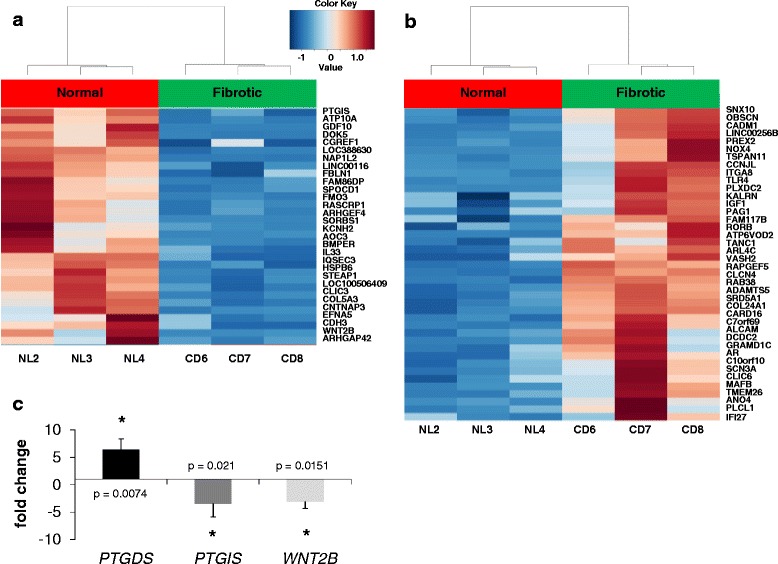
Differential gene expression profiles of HIF isolated from normal colons and Crohn’s Disease (CD) fibrotic colons. **a** Heatmap and hierarchical clustering dendrogram of transcript abundance from RNA-seq performed on HIF RNA from three normal colons and three CD fibrotic colons depicting differentially expressed genes with reduced expression in CD fibrotic colon compared to normal colon. *Red* represents up-regulation of the gene’s expression, and *blue* down-regulation. **b** Heatmap depicting differentially expressed genes with increased expression in CD fibrotic colon compared to normal colon. **c** Validation by RT-PCR of the fold change in *PTGDS*, *PTGIS*, and *WNT2B* mRNA levels in CD fibrotic versus control fibroblasts is shown (*n* = 7; *PTGDS p* = 0.0074, *PTGIS p* = 0.021, and *WNT2B p* = 0.015)

RNA-seq analysis identified the fibrosis-associated changes in the HIF transcriptome associated with changes in DNA methylation. Using established criteria for analyzing RNA-seq data from normal and fibrotic RNA, we found 72 genes that were differentially expressed (Benjamin-Hochberg adjusted *p* value <0.05, FDR < 5 %, Fig. 2a and Additional file 2). The consistency of gene expression within each group is evident from the hierarchical clustering dendrogram. Of the 72 differentially expressed genes, 31 were downregulated and 41 upregulated. Only three genes, toll-like receptor 4 (*TLR4*) [29], interleukin 33, (*IL-33*) [30], and insulin-like growth factor 1 (*IGF-1*) [31], have been previously reported or hypothesized to have functions in intestinal inflammation, IBD, or other fibrotic diseases [32, 33].

### *WNT2B*, *PTGIS*, and *PTGDS* are subject to differential expression and differential DNA methylation

**Fig. 3:**
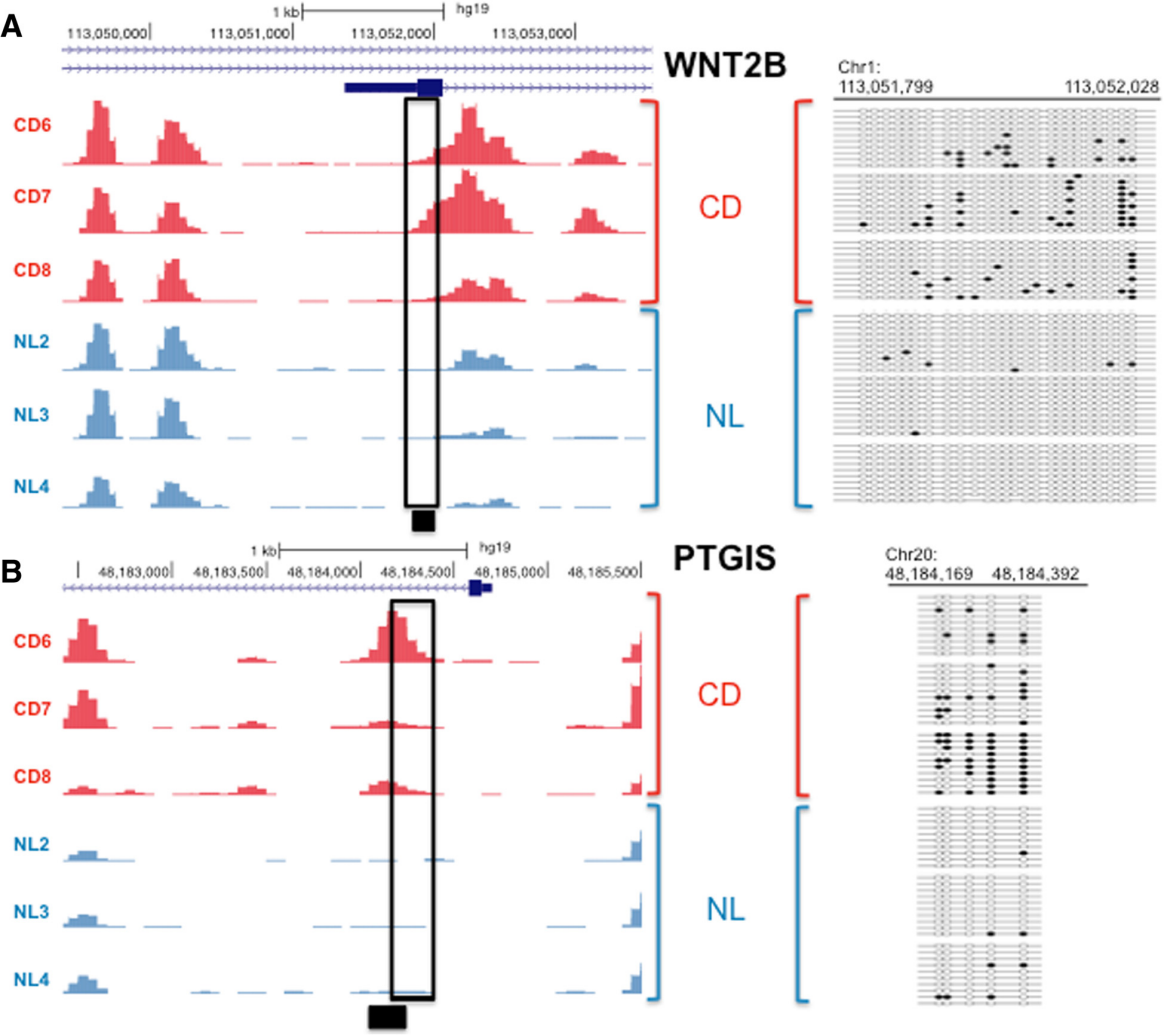
Bisulfite sequencing validation of differentially methylated *WNT2B* and *PTGIS* in fibrotic versus control HIF. **a** On the *left*, UCSC genome browser capture showing the MiGS read depth for three CD fibrotic (CD) and three control colon (NL) samples that shows the *WNT2B* promoter DMR and adjacent flanking regions. The range for the methylation counts (*y*-axis) for all samples in the UCSC genome browser was set at 0 to 150. The *solid black box* underneath the browser screen capture shows the location of the yes/no DMR region called from the MiGS data. On the *right*, targeted bisulfite sequencing validation of a region overlapping the DMR as indicated by the *rectangle* over the genome browser tracks. *Dark circles* indicate methylated and *open circles* unmethylated cytosines. Each row consists of a single sequenced clone. **b** MiGS data and targeted bisulfite sequencing illustrated as in (**a**) for *PTGIS*. The *y*-axis scale for all samples in the browser capture was set at 0 to 75 reads

We next examined which of the sharp yes/no DMRs in promoter regions that overlapped with genes were differentially expressed (Table 1). Promoter hypermethylation of two protein-coding genes, wingless-type mouse mammary tumor virus integration site family, member 2B (*WNT2B*) [34] and prostacyclin synthase (*PTGIS*) [35], were inversely correlated with alterations in their mRNA levels (Table 1). Validation by RT-PCR showed that the changes in *PTGIS* mRNA and *WNT2B* mRNA were decreased in fibrotic compared with normal HIF (Fig. 2b), consistent with the observed hypermethylation of each gene. In contrast, the mRNA level of prostaglandin D2 synthase (*PTGDS*), another member of the eicosanoid (prostaglandin) family [36], was increased in fibrotic HIF (Fig. 2b). Bisulfite sequencing was performed on the *WNT2B*, *PTGIS*, and *PTGDS* genes (Figs. 3a, b and Additional file 3) to validate the DMRs at base-pair resolution. The DMRs in the *WNT2B* and *PTGIS* promoters were confirmed to be hypermethylated (Fig. 3a, b). Bisulfite sequencing confirmed that *PTGDS* was hypomethylated in a 550-bp region spanning its promoter and coding sequence (Additional file 3). These findings corroborate the corresponding changes in expression and DNA methylation levels for all three genes (Additional file 3, Figs. 2b and 3a, b).

**Table 1:** Genes differentially DNA methylated and expressed in fibrotic HIF

Gene symbol	Hyper/hypo	Location of DMR	Fold change (B vs. A)	Adjusted *p* value
*WNT2B*	01	Promoter + 5′ UTR + exon + intron	0.19	0
*PTGIS*	01	Promoter + intron	0.18	0
*PTGDS* ^*a*^	10	Promoter + exon	6.44	0.01
*COL24A1*	*01*	Promoter + 5′ flank	5.94	0.01
*OBSCN*	*01*	Promoter + exon + intron	3.47	0.04
*RAB38*	*01*	Promoter + 5′ UTR + exon + 5′ flank	5.73	0
*ARL4C*	*01*	Intergenic + promoter + 5′ flank	3.43	0.04
*CLIC6*	*01*	Intergenic + promoter + 5′ flank	3.64	0.02
*FBLN1*	*01*	Intergenic + promoter + 5′ Flank	0.22	0
*KALRN*	*01*	Promoter + 5′ UTR + exon + intron + 5′ flank	3.64	0.03
*DCDC2*	*01*	Intergenic + promoter + 5′ flank	25.14	0.01
*KCNH2*	*01*	Promoter + exon + intron	0.13	0
*IFI27*	*10*	Promoter + 5′ UTR + exon + intron	4.06	0.04
*AOC3*	*10*	Promoter + 5′ UTR + exon + intron	0.04	1.16E−15
*ADAMTS5*	*10*	Promoter + intron	5.11	0

The genes that were found to be both differentially DNA methylated in their promoter regions and inversely correlated with gene expression are shown. Hyper/hypo column denotes whether genes are yes/no hyper- (01) or hypo- (10) methylated in fibrotic HIF. Italicized *01* and *10* represent genes that were quantitatively differentially DNA methylated and not analyzed further in this study. The location of the DMR, the fold change in mRNA expression detected by RNA-seq, and the adjusted *p* value are shown

^a^PTGDS fold change and *p* values are from RT-PCR validation

### Novel functional gene networks in Crohn’s disease-associated fibrosis

As a first step in determining how differential DNA methylation contributes to intestinal fibrosis pathogenesis, we used the GeneMANIA algorithm to predict network-based functional associations between genes differentially methylated and differentially expressed in fibrotic HIF (list of input genes in Additional file 4). The attributed functions fall into two main groups. The first is extracellular matrix structure and organization, which is highly relevant to fibrosis of the intestine and other fibrotic disorders [37, 38]. The second is guanine nucleotide exchange factor signaling, including the small GTP proteins RHO and RAC [39], previously described in gastrointestinal ulcer healing and in other fibrotic diseases but not in intestinal fibrosis [40, 41].

**Fig. 4:**
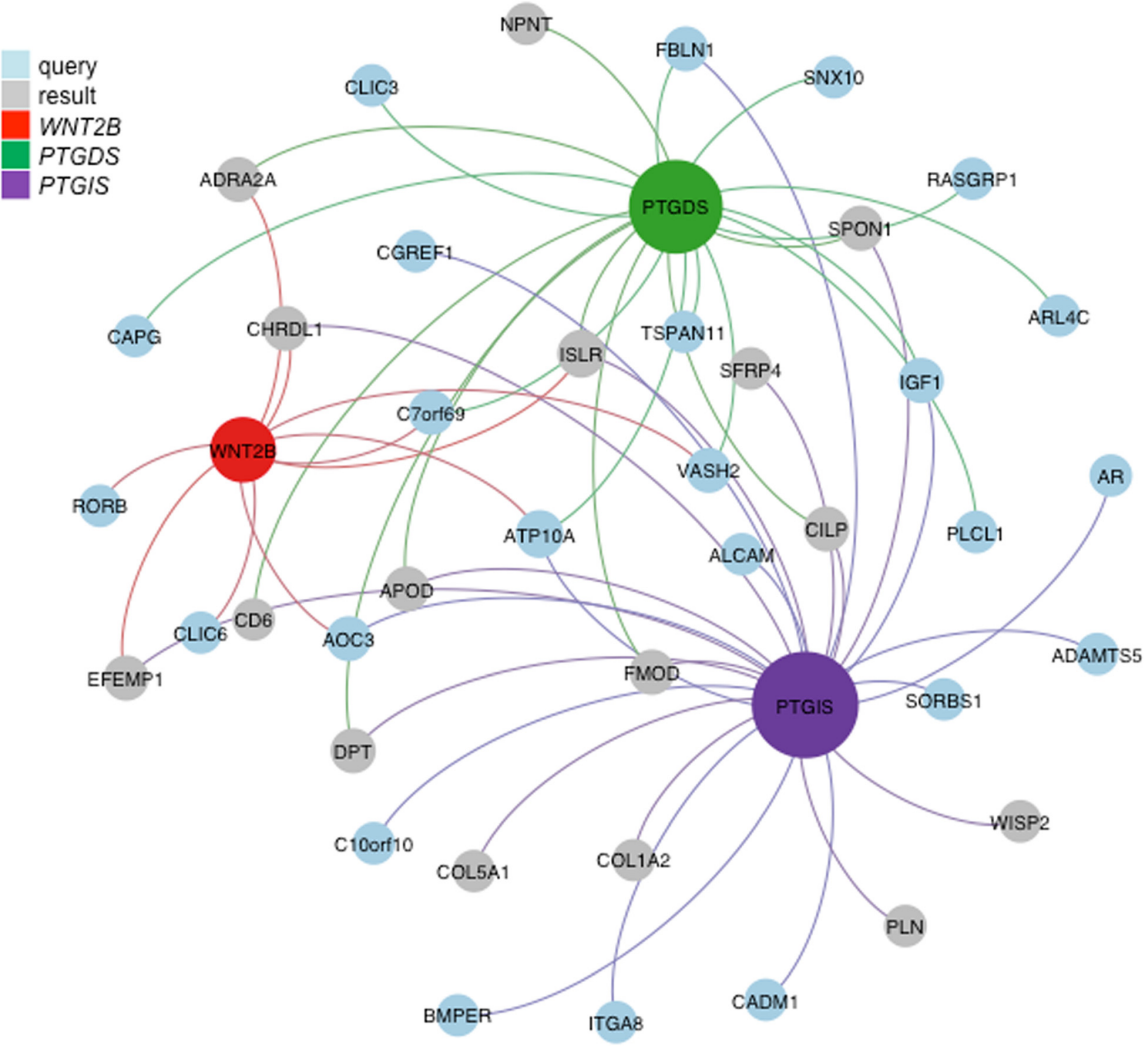
Interaction network for genes differentially expressed and differentially DNA methylated in fibrotic HIF. The *blue circles* represent the genes entered into the Cytoscape plugin for GeneMANIA. While the network was built for all differentially expressed genes, only the interactions from the subset that show both differential expression and DMRs are shown here. The *gray circles* are additional genes closely associated with the input genes. The size of the circle (node) is the number of neighbors each gene connects to. The edges are indicated by associations found through previously published co-expression, co-localization, and genetic and physical interactions. The genes/nodes with edges connected to *PTGDS* are colored *green*, the edges that connect to *PTGIS* are colored *violet*, and those to *WNT2B* are colored *red*

Sub-networks that include the three differentially methylated and expressed genes *WNT2B*, *PTGIS*, and *PTGDS* (Fig. 4) and the genes that interact with them were identified (Additional files 5, 6, and 7). *PTGDS* and *PTGIS* were associated with fibulin 1 (*FBLN1*) and *IGF-1*, both linked with extracellular matrix structure and fibrosis [31, 42]. *PTGIS* is also linked with other genes involved in the organization and structure of the extracellular matrix, for example, ADAM metalloprotease with thrombospondin motifs 5 (*ADAMTS5*) and the alpha 2 chain of type I collagen (*COL1A2*) [42–44]. There are also other interactions not previously described in any fibrotic disease; for example, both *PTGDS* and *WNT2B* are linked with vasohibin 2 (*VASH2*) [45] and chromosome 7 open reading frame 69 (*C7orf69*).

## Discussion

The objective of our studies was to obtain novel insights into the molecular mechanisms that underlie intestinal fibrogenesis. Through definition and integration of the DNA methylome and transcriptome, we have revealed functional candidate gene networks for three protein-coding genes: *WNT2B*, *PTGIS*, and *PTGDS*, which have not previously been described in CD or other fibrotic diseases. By employing the next generation sequencing-based approach, MiGS [24] and RNA-seq, we were able to achieve genome-wide coverage and improved accuracy over previous studies that have used microarrays to profile changes in DNA methylation and gene expression in inflammatory bowel disease and other fibrotic disorders.

Our experiments were performed in HIF purified from fibrotic or normal colon tissue to minimize the confounding effects of cell type heterogeneity of whole blood or tissue that have been widely used in previous epigenetic studies [28]. The large number, size, and genome-wide distribution of DMRs in fibrotic HIF suggest that this epigenetic modification has an important role in intestinal fibrosis. We observed sharp yes/no differences in DNA methylation throughout the genic and intergenic regions of the fibrotic HIF genome. Only a comparatively low percentage of DMRs occurred in promoters of genes and in CpG islands. The vast majority of DMRs were located within introns and intergenic regions. Intergenic sequences contain enhancers and insulators that are associated with regulation of gene expression during differentiation and organogenesis [7, 8]. Other distinct functions for DNA methylation have been proposed within introns and CpG island shores [28, 46, 47]. For example, DNA methylation in introns can modulate alternative exon splicing [46, 47] and in shores, differential DNA methylation is tissue-specific and may regulate transcription from alternative start sites [28].

The precise mechanism by which DNA methylation regulates gene transcription remains poorly defined. Methylated CpGs can prevent the binding of some transcription factors [7], and other evidence indicates that methylation of DNA can alter nucleosome occupancy and alternative polyadenylation of mRNA during transcription [48–50]. Whether de novo DNA methylation directly represses genes or whether gene silencing precedes or follows methylation is still debated, but the available evidence indicates that this will be dependent in large part on the genomic location of the methylated DNA sequence [7, 8]. This is a rapidly advancing area with new compelling evidence for causal associations between DNA methylation changes and phenotype [51, 52]. Our detection of DMRs provides a resource of candidate loci for future work to understand the role of DNA methylation in different genomic locations at different stages of pro-fibrotic gene expression.

We identified three genes: *WNT2B*, *PTGIS*, and *PTGDS* in which differential methylation of the DNA in each promoter region was inversely correlated with gene expression in fibrotic HIF. Complex roles for the WNT signaling pathway in the fibrosis of a number of organs including the lung and kidney and in controlling tissue homeostasis, cell proliferation, migration, differentiation, apoptosis, and organogenesis have been reported [53, 54]; however, this family of genes has not previously been associated with intestinal fibrosis. The decreased expression of hypermethylated *WNT2B* is a novel finding both in the context of fibrosis and CD. Interestingly, increased expression of *WNT2B* has been detected in the intestinal mucosa of UC patients [55], suggesting that *WNT2B* may perform distinct functions in CD and UC.

In the case of the prostaglandin family, central functions are well-established in homeostasis, inflammation, and other fibrosis disorders [56]. Profibrogenic functions for prostaglandin F2α and anti-fibrotic effects of prostaglandin E_2_ have been documented in lung fibrosis [57, 58]. In addition, the product of PTGIS catalysis, PGI_2_, has been shown to be anti-fibrotic in the lung [56] and also in a prostacyclin receptor null mouse model of cardiac fibrosis [59]. We found that *PTGIS* was hypermethylated and repressed in fibrotic HIF. Hypermethylation of the *PTGIS* promoter is a feature of colorectal cancer, but decreased expression of *PTGIS* has not previously been linked with IBD [60]. A further finding of our studies was that the lipocalin-type *PTGDS* gene was hypomethylated and its mRNA levels increased in fibrotic HIF. Our observations are supported by studies showing (i) increased levels of lipocalin type PTGDS in colitis, (ii) increased expression in UC patients in parallel with disease activity, and (iii) decreased DSS-induced colitis in L-PTGDS knockout mice [61]. Conflicting data in other studies indicate that in the kidney, PGD_2_, the product of PGDS catalytic activity, promotes the development of tubulointerstitial fibrosis [62], whereas a protective function of PGD_2_ has been reported in TNBS colitis [63]. These observations may reflect tissue-specific differences or methodological discrepancies between the aforementioned studies.

Based on previous studies including a recent study of fibrotic liver disease, the average age difference of 20 years between our fibrotic Crohn’s disease and normal patient groups is highly unlikely to be a confounding factor in the differential DNA methylation we identified [23]. Comparison of the DNA methylomes of dermal fibroblasts from individuals with a much greater disparity in age (<23 years old vs. >60 years old) showed a change in DNA methylation levels of >15 % at only 75 CpG sites, none of which were highly significant (*p* < 0.001) [64]. More importantly, none of these sites were differentially methylated in fibrotic HIF.

Studies in tissues such as the heart, and in diseases such as cancer, indicate that the same epigenetic pathways that are beneficial in organ development are perturbed in the fully developed or diseased tissue. The concept of antagonistic pleiotropy [65] suggests that key pathways are advantageous at early stages of development, e.g., in myofibroblast differentiation and wound healing, but in later life, these same pathways become detrimental as they promote myofibroblast activation and progressive fibrosis [65, 66]. Thus, the available evidence indicates that alterations in DNA methylation during development could serve as key determinants of the pro-fibrotic phenotype. Together with genetic and environmental factors, DNA methylation could be a major contributor to the progression of injury in CD and to the establishment of intestinal fibrosis. An important aim of future studies will be to determine changes in DNA methylation in patients with different stages of fibrostenotic CD. This could help stratify distinct subtypes of CD and identify those patients most likely to develop intestinal fibrosis

## Conclusions

Our detailed map of the methylome integrated with the transcriptome has revealed fibrosis-associated regulation of *WNT2B*, *PTGIS*, and *PTGDS*. The networks for these genes reveal interactions with other factors important in development and extracellular matrix synthesis. This suggests that key sites of differential DNA methylation can lead to the molecular aberrations that underlie IBD-associated fibrosis and provide potential targets for future development of prognostic strategies and specific anti-fibrotic therapies. Our data also provide a basis for defining the contribution of genome-wide changes in DNA methylation to the pathogenesis of fibrotic disorders.

## Methods

### Colon tissue and cell culture

Colon resection specimens were obtained from patients with CD-associated fibrosis and control colon specimens were obtained from histologically normal tissue from patients with diverticulitis (Additional file 8) under the approval of the Cleveland Clinic institutional review board (IRB 06-050). The approved protocol included a waiver of informed consent for redundant tissues obtained through the Cleveland Clinic Human Tissue Procurement Facility in accordance with Cleveland Clinic IRB policy. Tissue specimens were de-identified and made available following authorization by a Cleveland Clinic surgical pathologist. Human intestinal fibroblasts (HIF) were cultured from colon specimens and their purity confirmed as previously reported by established methods [67].

### DNA methylome profiling

Genome-wide DNA methylation was mapped using methyl-CpG binding domain-isolated genome sequencing (MiGS) [24], with the following differences. Genomic DNA (DNEasy kit, Qiagen) was extracted from normal and fibrotic cultured HIF. DNA (10 μg) was sheared on a Covaris S220 to an average size of 120 bp. Methylated DNA fragments were purified on PrepEase DNA columns (USB) and captured with the methyl-CpG binding protein MBD2 (MethylMiner Methylated DNA Enrichment kit #ME10025, Invitrogen). A sequencing library for each immunoprecipitated DNA sample was prepared using Ilumina’s ChIP-seq DNA Sample Prep Kit. The quality of the DNA was assessed on an Agilent Bioanalyser. Sequencing libraries of methylated DNA fragments were analyzed and next generation sequencing performed on an Ilumina HiSeq 2000. Read lengths of 50 bp were sequenced at an average depth of 161,226,405 reads (minimum: 130,528,176 reads, maximum: 190,943,105 reads). Reads were aligned to assembly *hg19* of the human genome (Genome Reference Consortium Human Build 37) using the *bowtie2* short read alignment software (options: -N 1 -L 20 --phred33) [68].

### Bioinformatic analysis of differential DNA methylation

Differential DNA methylation was detected using the integrated signal deconvolution, pattern recognition, and differential testing (*iDPT*) framework [69] (https://idpt.github.io/dptscan/). Using this framework, reads were extended to the average fragment length. Average coverage (e.g., averages of the per-base coverage rounded to integers) was computed within 50-bp non-overlapping windows tiling the entire genome. Initial filtering removed all windows where no sample had a read count above a false discovery threshold established by a Poison distribution that assumed an even coverage of reads across the genome. Signal deconvolution was performed using a Bayesian mixture of three Poisons model, producing a probability of methylation for each window in each sample. Pattern recognition used these probabilities and a scan statistic to produce regions of consistent methylation status, allowing for small gaps.

Finally, differential testing was performed using a linear mixed-effects model and an output table that contained fold changes, and multiple testing-adjusted *p* values for each DMR was produced. The output differentiated between sites showing a sharp yes/no methylation difference (defined as all samples in one group are predicted by the model to have no methylation, and all samples in the other group are predicted to be methylated) versus sites that show a quantitative difference in reads (the model predicts all samples across all groups to be methylated, but the read counts show a statistically significant difference). Only the sharp sites were used for subsequent analysis because they are more likely to represent differential DNA methylation rather than other phenomena that can change read counts, such as copy number variation.

The R package *goldmine* was used to analyze the genomic context of the detected regions with respect to known genes and features (http://jeffbhasin.github.io/goldmine). The *goldmine* functions, *getCpgFeatures*, *getFeatures*, and *getGenes* were used to obtain CpG islands, ENCODE supertracks, and RefSeq gene models from the database of the UCSC Genome Browser [70, 71]. This data was used by the *goldmine* function to provide detailed annotation of how these datasets relate to the regions.

### RNA sequencing

For RNA sequencing (RNA-seq), RNA was extracted from control and fibrotic HIF using the RNEasy mini kit (Qiagen). A gene sequencing library for each sample (1 μg RNA) was prepared using the TruSeq™ RNA Sample Prep Kit v2. Next generation sequencing was performed on an Illumina HiSeq 2500. After quality assessment, reads were aligned to *hg19* using GSNAP [72]. Read lengths of 100 bp were sequenced at an average depth of 53,390,000 reads (minimum 39,470,000 reads, maximum 62,550,000 reads). Only reads that aligned to a single locus in the *hg19* genome assembly were retained for further analysis. Read counts for RefSeq genes were computed using HTSeq-count program [73]. Differential expression analysis was performed on this count table using the negative binomial test provided by the DESeq package [74] in the R statistical computing environment (*http://www.R-project.org/*). Transcripts with *p* values less than 0.05 after adjustment by the Benjamini-Hochberg procedure were considered statistically significant.

### Bisulfite sequencing

For validation of DMRs detected by the genome-wide DNA methylome analysis, genomic DNA (330 ng) was prepared using the DNEAsy kit (Qiagen). Bisulfite conversion of the DNA was performed with the EZ DNA Methylation-Lightning Kit #D5030 (Zymo). Bisulfite-converted DNA (1 μl) was amplified by PCR with iTaq DNA polymerase (BioRad), and primers were designed using the MethPrimer website (see Additional file 9). DNA was cloned (Topo TA cloning kit, K4575-01, Invitrogen), and the cloned DNA (prepared using PureLink Miniprep kit, Invitrogen) was digested with EcoR1 to verify cloning of the inserted DNA. DNA from at least ten different clones was then sequenced at the Genomics Core of Cleveland Clinic’s Lerner Research Institute. DNA methylation of individual CpG sites was analyzed using Quma software (http://quma.cdb.riken.jp/).

### Reverse transcriptase PCR (RT-PCR)

For validation of changes in mRNA levels of differentially DNA methylated genes, total RNA was extracted from fibrotic and normal HIF and reverse transcribed, and complementary DNA (cDNA) was amplified by RT-PCR. Five microliters of cDNA was amplified in the presence of 0.125 μmol/L each of the 5′ and 3′ primers (Biosynthesis, Lewisville, TX) and 1 U of Taq DNA polymerase (Roche, Mannheim, Germany). PCR was performed in a DNA thermal cycler using pre-optimized temperatures and times, and the primers (listed in Additional file 9) were used to quantify mRNA levels. Fifteen microliters of the PCR product were subjected to electrophoresis on 1.5 % agarose gel and stained with 0.5 μg/ml ethidium bromide, using 100 bp DNA ladder as a marker.

### GeneMANIA network analysis

The GeneMANIA algorithm (version 3.2.1, http://www.genemania.org) employs functional interaction data to create a network containing putative functional links between genes. We used the GeneMANIA Cytoscape plugin [75] to generate a network for the genes identified in this study based on protein-protein interactions, genetic interactions, and co-expression profile databases. Data on shared protein domains and co-localization were eliminated from the analysis, to minimize the number of false positives. A sub-network was extracted by restricting the network to only nodes that interact with defined genes of interest and was plotted using Gephi (https://gephi.github.io/).

### Availability of supporting data

The DNA methylation and RNA-seq data sets (raw and normalized) supporting the results of this article are available in the NCBI Gene Expression Omnibus (GEO) repository: http://www.ncbi.nlm.nih.gov/geo/, accession number GSE67250.

## Additional files

Additional file 1: Table S1. Differentially methylated regions (DMRs) between normal and fibrotic HIF. Each row represents one DMR. For each DMR, a unique ID, coordinates in the form of chr, start, end (hg19), and the DMR width are provided. Each DMR is categorized by a pattern code: “01” represents hypermethylation in fibrotic samples, and “10” represents hypomethylation in fibrotic samples. The “ratio” column represents the magnitude of the difference in sequencing signal as a log2 fold change, “p.value” is the unadjusted *p* value, and “q.value” is the Benjamini-Hochberg adjusted *p* value. “Genomic_context” specifies if the DMR falls within a portion of an annotated gene model. Multiple overlaps are resolved using the priority: promoter > exon > intron > 3′ end > intergenic. Overlap with CpG shores, islands, and shelves is provided, along with the distance to the nearest gene and the names of the nearest genes. (XLS 461 kb) Additional file 2: Table S2. RNA-seq analysis of differentially expressed genes in control and fibrotic HIF. Total mRNAs from three different normal (2, 3, 4) and fibrotic fibroblasts (6, 7, 8) were subjected to RNA-seq analysis. Normalized FPKM values were averaged and the log base twofold change for fibrotic versus normal HIF calculated. FDR adjusted *p* values are shown. Genes were considered significant if the adjusted *p* value was <0.05 and the log fold change was at least 1.5-fold. The differentially expressed genes are highlighted. (XLS 45 kb) Additional file 3: Figure S1. Bisulfite sequencing validation of differentially methylated *PTGDS* in fibrotic versus control HIF. UCSC genome browser capture (left panel) of regions of differential DNA methylation for *PTGDS* from MiGS are shown next to the corresponding region of the gene validated by bisulfite sequencing (right panel). Three CD fibrotic (top panel) and three control (NL, lower panel) samples are shown. Dark circles indicate methylated and open circles unmethylated cytosines. Each row consists of a single sequenced clone. The range for the methylation counts (*y*-axis) in the UCSC genome browser was set at 1 to 128. (TIFF 1517 kb) Additional file 4: Table S3. List of input genes for GeneMANIA. The list of genes used to construct an interaction network in GeneMANIA in order to obtain functional enrichment is provided. A subset of the identified interactions are shown in Fig. 4. (XLSX 9 kb) Additional file 5: Table S4. Network gene associations for *WNT2B.* List of genes in RNA-seq data set (bold italics) and in GeneMANIA database (italics) that interact with *WNT2B* are shown. (XLSX 45 kb) Additional file 6:Table S5. Network gene associations for *PTGIS*. List of genes in RNA-seq data set (bold italics) and in GeneMANIA database (italics) that interact with *PTGIS* are shown. (XLSX 45 kb) Additional file 7:Table S6. Network gene associations for *PTGDS*. List of genes in RNA-seq data set (bold italics) and in GeneMANIA database (italics) that interact with *PTGDS* are shown. (XLSX 41 kb) Additional file 8: Table S7. Patient characteristics. The characteristics of the patients with Crohn’s disease and controls from whom colon specimens were obtained for the DNA methylome and RNA Seq analyses are shown. (XLSX 9 kb) Additional file 9: Table S8. Primers for gene expression and bisulfite sequencing. The bisulfite sequencing primers for specific chromosomal locations and the qRT-PCR primer sequences and accession numbers, for *WNT2B*, *PTGIS*, and *PTGDS* are shown. (XLSX 11 kb)

## Abbreviations

CD: Crohn’s disease
CGIs: CpG islands
DMRs: differentially methylated regions
ECM: extracellular matrix
FBLN1: fibulin-1
FDR: false discovery rate
HIF: human intestinal fibroblasts
IBD: inflammatory bowel disease
IGF-1: insulin-like growth factor 1
MBD: methyl-CpG binding domain
MiGS: MBD-isolated genome sequencing
PTGDS: prostaglandin D2 synthase
PTGIS: prostacyclin synthase
UC: ulcerative colitis
VASH2: vasohibin 2
WNT2B: wingless-type mouse mammary tumor virus integration site family, member 2B
