# Supplementary figures and images for: Genome-wide analysis of DNA methylation and gene expression defines molecular characteristics of Crohn’s disease-associated fibrosis
Source: Clin Epigenetics. 2016 Mar 12;8:30. doi: 10.1186/s13148-016-0193-6 (PMC4789277; doi:10.1186/s13148-016-0193-6)

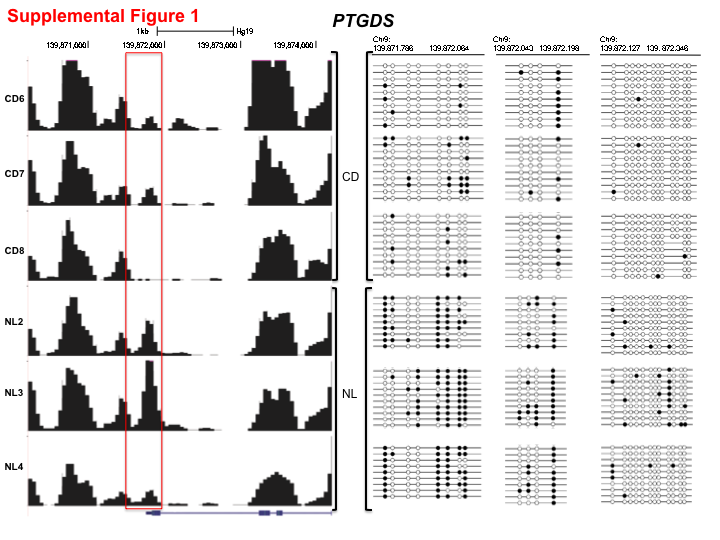

Supplement: Additional file 3: Figure S1. — Bisulfite sequencing validation of differentially methylated PTGDS in fibrotic versus control HIF. UCSC genome browser capture (left panel) of regions of differential DNA methylation for PTGDS from MiGS are shown next to the corresponding region of the gene validated by bisulfite sequencing (right panel). Three CD fibrotic (top panel) and three control (NL, lower panel) samples are shown. Dark circles indicate methylated and open circles unmethylated cytosines. Each row consists of a single sequenced clone. The range for the methylation counts (y-axis) in the UCSC genome browser was set at 1 to 128. (TIFF 1517 kb) [file 13148_2016_193_MOESM3_ESM.tiff]
